# Sexuality and Intimacy in Inflammatory Bowel Disease: A Phenomenological Study

**DOI:** 10.3390/healthcare14040526

**Published:** 2026-02-19

**Authors:** Caterina Mercuri, Vincenzo Bosco, Vincenza Giordano, Teresa Rea, Raúl Juárez-Vela, Patrizia Doldo, Silvio Simeone

**Affiliations:** 1 Department of Clinical and Experimental Medicine, Magna Graecia University, 88100 Catanzaro, Italy; doldo@unicz.it (P.D.); silvio.simeone@unicz.it (S.S.); 2 Department of Medical and Surgical Sciences, University Hospital Mater Domini, Magna Graecia University, 88100 Catanzaro, Italy; vincenzo.bosco@unicz.it; 3 Department of Public Health, University of Naples Federico II, 80131 Naples, Italy; vincenza.giordano@unina.it (V.G.); teresa.rea@unina.it (T.R.); 4 Faculty of Health Sciences, University of La Rioja, 26006 Logroño, Spain; raul.juarez@unirioja.es

**Keywords:** Inflammatory Bowel Disease, sexual health, qualitative research, patient experience, intimacy, quality of life

## Abstract

**Background/Objectives:** Inflammatory Bowel Disease (IBD) often occurs during early adulthood and substantially affects physical, psychological, and relational well-being. Although sexual health is a fundamental component of quality of life, it is rarely addressed in clinical practice and remains insufficiently explored in research. This study aimed to explore the lived experiences of individuals with IBD regarding sexuality and intimate relationships. **Methods:** Qualitative phenomenological design was adopted. Nineteen adults with a confirmed diagnosis of Crohn’s disease or Ulcerative Colitis were purposively recruited from a gastroenterology and endoscopy unit of a university hospital in Southern Italy. Data were collected through in-depth, audio-recorded interviews conducted in Italian, transcribed verbatim, and analyzed using Cohen’s phenomenological method. Lincoln and Guba’s criteria were applied to ensure methodological rigor. **Results:** Five main themes and two subthemes emerged. Participants reported that IBD profoundly affected their sexual lives, not only through physical symptoms but also by eliciting emotional distress and avoidance behaviors. Stigmatization of symptoms such as incontinence and bloating frequently led to withdrawal from physical intimacy. Changes in body image, including weight fluctuations, scarring, and fear of a possible stoma, were associated with feelings of shame and self-alienation. Sexuality was often described as mechanical and emotionally detached, although some participants reported processes of relational reconnection. Concerns about relationship stability and uncertainty about the future were common, alongside a persistent lack of communication with healthcare professionals regarding sexual health. **Conclusions:** Sexual health in people with IBD is essential yet frequently overlooked. A holistic and empathetic approach that integrates sexual health into routine IBD care may enhance emotional well-being, improve partner communication, and strengthen the overall quality of care.

## 1. Introduction

Inflammatory Bowel Disease (IBD) represents a group of diseases characterized by persistent inflammation of the gastrointestinal tract, with Crohn’s disease (CD) and Ulcerative Colitis (UC) being the predominant forms [1,2]. Crohn’s disease can affect any part of the digestive system, with characteristic transmural and discontinuous inflammation.

In contrast, Ulcerative Colitis is limited to the mucosa of the colon and rectum, with continuous inflammation [3,4].

The pathogenesis of Inflammatory Bowel Disease (IBD) is widely recognized as multifactorial, resulting from a complex interaction among genetic susceptibility, immune dysregulation, environmental factors, and alterations in the gut microbiota. Although the precise etiology remains unclear, these factors act synergistically in influencing disease onset, progression, and severity. Genetic susceptibility contributes to IBD pathogenesis through variants involved in immune regulation and host–microbiota interactions, as shown by genome-wide association studies identifying risk loci such as NOD2, ATG16L1, and IL23R, particularly in Crohn’s disease; however, genetic factors alone are insufficient to initiate the disease [5].

Currently, IBD affects more than 10 million people globally, with an estimated 3.2 million cases documented in Europe and more than 2 million in North America. The prevalence is rapidly increasing, particularly in newly industrialized countries in Asia, the Middle East, and South America, where these conditions were historically less prevalent [6,7,8].

IBD typically follows an unpredictable course, characterized by alternating phases of remission and exacerbations [9,10]. The symptomatology of IBD is broad and variable [11,12]. Additionally, many individuals with IBD develop extraintestinal manifestations, [13,14]. More severe complications may include stenosis, fistulas, infections, and, in the long term, an increased risk of colorectal cancer [15,16,17].

Beyond its physical manifestations, the disease significantly impacts both social and occupational dimensions, reducing participation in daily activities and productivity, thereby contributing to decline in quality of life [18,19,20,21].

The chronicity of the disease, coupled with its unpredictability and the prevailing social stigma, often leads to experiences of shame, insecurity, and isolation, particularly among younger patients [22,23,24]. IBD is predominantly diagnosed between the ages of 20 and 40, a critical developmental stage characterized by significant changes in body image, the establishment of intimate relationships, and anticipation for parenthood [25,26,27,28]. Within this vulnerable life phase, negative perceptions of one’s body, uncertainty about one’s health, and the economic burden of the disease significantly contribute to heightened emotional distress, which consequently exerts a profound impact on sexual health and intimacy [29,30,31,32,33].

Sexual health is a fundamental element of an individual’s overall well-being, profoundly affecting quality of life [34]. The World Health Organization (WHO) defines it as a state of physical, emotional, mental and social well-being in relation to sexuality, and not simply the absence of disease or dysfunction [35].

Nearly 50% of patients report a negative impact on their sexual health, manifested in the form of decreased libido, dissatisfaction, relationship dissolution, or challenges in communicating with their partners [36,37,38,39]. Since sexuality is a key determinant of quality of life, particularly among younger individuals, concerns related to sexual function and intimacy rank among the most pressing issues reported by IBD patients [40,41]. In this context, a number of studies, including those conducted by Timmer [42,43], have consistently highlighted the compromise of sexual health in individuals with IBD, emphasizing how this condition significantly affects patients’ overall well-being and relational lives. IBD’s impact on sexual health is driven by a complex interplay of interconnected factors. Among these, alterations in body image play a central role: surgical scars, fluctuations in body weight, and the presence of ostomies can adversely impact self-perception, engendering feelings of shame and inadequacy [30,44]. Such physical changes are often accompanied by side effects of drug treatments (such as steroids or antidepressants), pain during intercourse, and performance anxiety, which make the sexual experience a source of discomfort rather than pleasure, particularly during periods of disease activity [45,46]. Women more frequently report discomfort related to physical appearance and fertility, while men focus on problems related to sexual function, with the prevalence of erectile dysfunction reaching 45% in male patients with IBD [47,48,49,50,51].

Despite recognizing sexuality as a key component of their quality of life, patients often find it difficult to engage in open dialogue with healthcare professionals, who may avoid the topic altogether: both men and women report the rarity of conversations about sexual health within health care facilities, often avoided out of embarrassment or the belief that they are not prioritized over clinical disease management [52,53]. The cumulative effect of these difficulties negatively affects patients’ relational and emotional quality of life, underscoring the urgency of a more holistic and inclusive approach. Although sexual health is an integral part of overall well-being [54], its exploration within the context of IBD remains markedly underdeveloped. The existing literature is sparse and fragmented, exposing substantial gaps in both clinical practice and scientific research.

Therefore, the present qualitative study aims to describe lived experiences of subjects with IBD concerning their sexual health and intimate relationships. Understanding this under-investigated aspect in depth can help develop more sensitive and integrated interventions, improving the overall care and well-being of people with IBD.

## 2. Materials and Methods

### 2.1. Design

The study was conducted by the phenomenological hermeneutic approach developed by Cohen [55], which combines features of Husserlian descriptive phenomenology and Gadamerian interpretive phenomenology [56].

While descriptive phenomenology aims to reveal the essence of a phenomenon by bracketing external influences, interpretive phenomenology focuses on understanding and interpreting the lived experiences of individuals within their specific social, cultural, and political contexts [57,58]. This methodological framework facilitates an in-depth examination of subjective experiences and the significance ascribed to those experiences by participants [59,60].

By prioritizing the participants’ perspectives and focusing on meaning-making, this approach provides valuable insights into the intricacies of lived experiences. For these reasons, it is considered particularly appropriate for nursing research, especially when investigating underexplored or sensitive topics [55].

### 2.2. Sample

Purposive sampling was adopted for participant recruitment. The subjects were contacted as they were undergoing treatment by the Gastroenterology and Endoscopy Operative Unit of the “Renato Dulbecco” University Hospital of Catanzaro. Enrollment was conducted during the period from May 2025 to November 2025. All participants had an established diagnosis of IBD and were already receiving standard medical therapy at the time of the interview. The qualitative phase was intentionally scheduled to begin after the completion of preparatory organizational and coordination activities with the clinical units involved, which were carried out following ethics approval.

To be included in the study, potential participants had to meet the following inclusion criteria:

(a) Being at least 18 years of age;
(b) Ability to speak and understand the Italian language;
(c) Willingness to voluntarily participate in the study;
(d) Confirmed diagnosis of IBD.

Exclusion criteria included:

(a) Not being of age according to Italian law;
(b) The presence of severe pre-existing cognitive impairments (e.g., dementia);
(c) Diagnosis of major psychiatric disorders;
(d) Inability to provide valid informed consent and voluntary withdrawal from the study.

The objectives and details of the study were explained by a researcher to each participant before informed consent was obtained. The ability to withdraw consent at any time was ensured, as well as the confidentiality of personal data throughout all phases of the study. Patients who had expressed interest in participating and provided their contact information were subsequently contacted by telephone. During the call, a detailed explanation of the project was provided and an interview appointment agreed upon. The date and setting of interviews was determined according to the individual availability of the participants. Once the informed consent was obtained, each participant was assigned an alphanumeric code to preserve his or her anonymity.

### 2.3. Data Collection

Following the phenomenological methodology adopted, the first step for all researchers involved in the study was bracketing [55], or the intentional suspension of personal preconceptions and beliefs regarding the phenomenon under investigation. This technique, also referred to as critical reflection, consists of researchers noting their assumptions, prior experiences, intuitions, and cultural references to avoid subjective interference in data analysis [61,62].

In line with Cohen’s hermeneutic phenomenological approach, bracketing was not understood as a full suspension of pre-understandings (typical of Husserlian descriptive phenomenology), but as a process of critical self-awareness. This interpretation is consistent with Cohen [55], who describe bracketing as the documentation and acknowledgment of assumptions rather than their elimination. This reflective stance supported the interpretive phase by allowing researchers to engage in a dialogical process between participants’ narratives and their own pre-understandings, consistent with Gadamerian hermeneutics. Reflexivity was maintained through written memos, team discussions, and an audit trail documenting interpretive decisions, as recommended by Lopez & Willis [63]. Bracketing was maintained throughout the research process, helping to ensure methodological rigor and focus the analysis exclusively on the participants’ point of view. The interviews were conducted by two researchers (CM and VG), who had had no previous contact with the participants. After explaining the nature of the study clearly and understandably, each participant independently chose the location and time of the interview, favoring familiar and comfortable settings, particularly their homes, to encourage free and spontaneous storytelling, free of external conditioning. The interviews were conducted in the native language of the participants and researchers (Italian), and began with a single open-ended question:

“Can you describe how your experience with IBD has affected your intimacy, sexuality, or relationship?”.

This approach allowed participants full freedom of expression, making their “world” the central object of inquiry, rather than guiding the conversation toward predefined themes [64].

Although the interview opened with a single broad question, the data collection process did not rely solely on this initial prompt. Consistent with phenomenological interviewing principles, the researchers used non-directive, open follow-up techniques to facilitate depth and richness in participants’ narratives. Phenomenological prompting was employed when appropriate (e.g., “can you tell me more about that?”, “what was that like for you?”, “how did you experience that moment?”), in line with established guidance [65,66].

Reflective listening, paraphrasing, and the intentional use of silence were used to encourage elaboration without imposing predefined categories, as recommended in hermeneutic phenomenology [67]. Clarification questions were asked only when necessary to ensure accurate understanding of participants’ meanings. The absence of a structured interview guide was intentional and aligned with Cohen’s hermeneutic phenomenology, which prioritizes participant-led meaning-making and the dialogical unfolding of experience [55].

In keeping with the phenomenological approach, no additional structured questions were asked: the researchers adopted an empathetic and welcoming attitude, aimed at fostering a climate of trust and openness [68]. During the interview, special attention was paid to nonverbal aspects, body language, tone of voice and environmental context [69], elements that enriched understanding of the narrative.

At the end of the spontaneous narrative, participants were asked if they wished to add any further items. The interview ended only when the participant stated that he or she had nothing more to say. All interviews were audio-recorded, with participants’ consent, and lasted between 30 and 50 min. Data saturation was defined prospectively as the point at which no new meanings, nuances, or experiential variations emerged from subsequent interviews. This understanding aligns with phenomenological guidance describing saturation as the moment when additional accounts no longer deepen or expand the essence of the lived experience [55,70]. As the analysis progressed, the research team observed that after the nineteenth interview, participants’ narratives no longer introduced additional interpretive elements relevant to the phenomenon under study. At that stage, themes appeared stable and sufficiently rich to capture the complexity of participants’ lived experiences. This decision was supported by repeated readings, team discussions, and the convergence of interpretive insights across researchers, consistent with recommendations for determining saturation in phenomenological inquiry [71].

### 2.4. Data Analysis

Interviews were transcribed verbatim and supplemented with field notes. Each transcript was read multiple times, with the initial reading providing an overview of the participants’ experiences. This phase is described by Cohen as sometimes called “data immersion”. The goal of this immersion is to develop an initial understanding of the data that will guide subsequent coding during later analysis stages. The researchers then reread the transcripts line by line, assigning indicative themes to different passages.

Each transcript was independently reviewed by multiple members of the research team, who compared their interpretive notes and discussed emerging meanings until a shared understanding was reached.

This multi-researcher analysis reflects investigator triangulation, as recommended by Cohen [55], and ensured that themes were grounded in multiple perspectives rather than a single interpretive lens. All analytic decisions, including the evolution of codes, the consolidation of themes, and the rationale for each step, were documented in an audit trail, consistent with qualitative rigor guidance [70].

Approximately three weeks after the initial interview, participants were invited to a follow-up meeting where a concise summary of emergent themes and selected quotations was presented. Participants were asked whether the themes accurately reflected their lived experience, whether any important aspect had been overlooked, and whether the wording or conceptual framing of the themes felt appropriate. This dialogical form of member checking is consistent with hermeneutic phenomenology [67].

While no new themes emerged, some participants suggested minor adjustments to the wording of the themes, which were incorporated to enhance clarity and resonance. The absence of major discrepancies strengthened the credibility of the findings.

### 2.5. Rigor

Scientific rigor in qualitative research refers to the trustworthiness and validity of the study findings, ensuring that the research process is transparent, credible, and reliable. In this study, rigor was ensured by adhering to the criteria established by Lincoln and Cuba [72], which guarantees scientific rigor.

Credibility was strengthened through investigator triangulation: multiple researchers independently analyzed each transcript, compared interpretive notes, and discussed emerging meanings until a shared understanding was reached, in line with recommendations for hermeneutic phenomenology [55]. An audit trail was maintained throughout the analytic process, documenting how initial codes were developed, merged, or refined into themes, and providing full traceability of interpretive decisions [70].

Member checking was conducted approximately three weeks after the first interview. Participants reviewed a concise synthesis of the emergent themes and selected quotations and were asked whether these themes accurately reflected their lived experience, whether any important aspect had been overlooked, and whether the wording or conceptual framing felt appropriate. Minor suggestions regarding phrasing were incorporated, while no substantive discrepancies emerged. This dialogical form of participant validation aligns with hermeneutic phenomenology, which emphasizes co-interpretation and iterative refinement of meaning [67].

Dependability was ensured by transparent documentation of all methodological steps, while confirmability was supported through both the audit trail and the use of bracketing as critical self-reflection to minimize subjective interference. Transferability was facilitated by providing a detailed description of the study context and participants’ experiences, enabling readers to assess applicability to other settings.

After the scientific report was written, a structured translation and back-translation process was conducted in accordance with WHO methodology for cross-cultural and interlingual validation [73].

All interviews were conducted and analyzed in Italian. The initial translation of participants’ quotations into English was performed by two bilingual members of the research team, both native Italian speakers with advanced academic English proficiency and prior experience in qualitative health research. To ensure conceptual accuracy, an independent bilingual translator with expertise in health-related translation carried out the back-translation into Italian.

The research team then compared the original Italian quotations, the English translations, and the back-translated versions. Any discrepancies were discussed collaboratively until full conceptual equivalence was achieved. Particular attention was devoted to culturally embedded expressions, metaphors, and idioms. In these cases, literal translation was avoided when it risked distorting meaning; instead, priority was given to preserving the experiential and emotional nuance of participants’ narratives, in accordance with WHO recommendations for cross-cultural translation.

**Figure 1:**
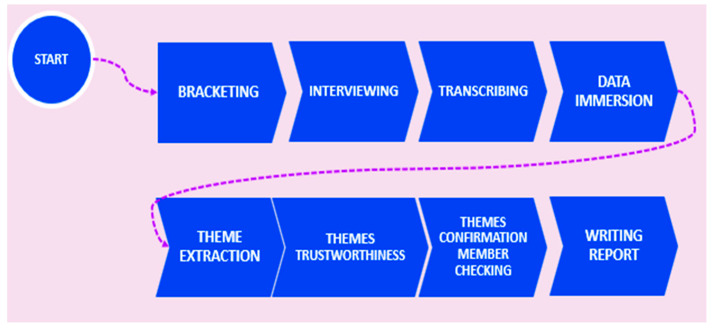
Phases of the study’s methodological process.

The stages of the methodological process are schematized in Figure 1.

### 2.6. Ethical Considerations

Ethical approval for this study was obtained from the Calabria Regional Center Ethics Committee on 23 May 2024, under Number 162.

The study was conducted following the guidelines of the Declaration of Helsinki. Participants were thoroughly informed about the aims and procedures of the study, assured of the confidentiality of their data, and asked to provide written informed consent. They were also informed that they could withdraw from the study at any time point. To ensure privacy and confidentiality during home-based interviews, conversations were conducted in a private room chosen by the participant, without the presence of family members or other individuals. All audio recordings and transcripts were stored on secure, password-protected institutional servers accessible only to the research team. The interviewer was trained to monitor signs of emotional discomfort and to pause, redirect, or discontinue the interview if needed. Participants were reminded that they could skip any question or stop the interview at any time.

## 3. Results

The study sample comprised 19 participants with Inflammatory Bowel Disease, diagnosed with Ulcerative Colitis or Crohn’s disease. Age ranged from 25 to 55 years, with an average age of about 41 years. The group included 10 women and 9 men. The majority of participants were married (84%), and the time since their diagnosis varied from 2 to 31 years, with an average of approximately 17 years. Main socio-demographic characteristics of participants are reported in Table 1. The table also includes the clinical information available for all participants (diagnosis and years since diagnosis), which provides a basic clinical framework for interpreting their narratives.

### 3.1. Overview of the Findings

The analysis revealed five principal themes and two subthemes that together form a coherent and interconnected representation of participants’ experiences. These themes reflect a continuum of bodily, emotional, and relational challenges, rather than separate or isolated dimensions. Table 2 summarizes the thematic structure, which is then presented in detail below:

1. Impact of the disease on sexuality.
  Subtheme: Stigmatization of physical symptoms leads to sexual avoidance.
2. Body image changes.
3. Being an automaton.
  Subtheme: Reconnection.
4. Uncertainty about the relational future.
5. Lack of communication with healthcare professionals.

Theme 1: Impact of The Disease on Sexuality

All participants explicitly acknowledged sexual health as a fundamental dimension of couple life, emphasizing its importance for emotional and relational well-being. They all clearly and thoroughly described how the illness affects this area, not only on a physical level, but even more so on a psychological one. Sexual health emerges as a complex and multifactorial phenomenon, deeply intertwined with the subjective experience of illness and its emotional and relational effects.

*HS08*: “it is a fundamental aspect of a couple’s relationship, no use denying it, always has been and always will be… this disease undermines the foundations of a relationship… because it not only affects the physical (open hand brought to the abdomen), the body, but also the head (open hand brought to the head; middle and ring index fingers strike hard above the forehead, above the right eye)”.

*KP 11*: “You can’t help but think about what was or what could be… even in those moments your mind doesn’t experience them, doesn’t enjoy the moment, but always thinks about them…”. This disease takes over your body and soul, steals your mind (tears in the eyes, shoulders forward, hands clasped in each other with open palms and facing upwards, shoulders tighten, gaze falls downwards and tears wet face and hands)”

Sub-theme: Stigmatization of physical symptoms leads to sexual avoidance

The data analysis showed a negative perception and stigmatization of the physical symptoms associated with the disease. These symptoms, often perceived as “disgusting” or “embarrassing,” generate a strong sense of shame that profoundly affects how patients experience their sexuality and intimacy.

The collected narratives demonstrate that such stigmatization often results in avoiding physical and sexual contact, creating a vicious cycle of emotional and relational isolation.

*BY 02*: “…in short, my symptoms are disgusting, they make me feel ashamed… I feel embarrassed even to sleep next to my partner sometimes…”

*EV 05*: “Your body isn’t working the way it should, and you don’t know what to do…. I feel so ashamed, embarrassed, and sometimes I prefer to avoid physical contact for this very reason”…”

Theme 2: Body Image Changes

Building on the emotional and relational disruptions described above, participants also reported profound changes in how they perceived their bodies.

Altered body image was a theme reported by almost all participants who constantly compared their bodies with those before diagnosis. This self-perception is often accompanied by feelings of estrangement and loss of physical identity, as the bodily changes caused by the disease and its treatments foster a view of one’s body as an alien entity, something no longer recognizable and constantly reminding them of the illness.

*BY 02*: “Every day I get more bloated, less attractive… and when I think that there could be a purse on my belly (turns head to the left, tilts chin and eyes upwards, eyelids blink rapidly, voice trembles and tears crease my face) I think that no one will ever be able to find me attractive, because I know it, I think it.

*JQ 10*: “I don’t recognize this body that always, every time, seems to remind me that it doesn’t work… when I think about what it could be… I get shivers, fear… in short, I imagine in those moments a constant flatulence…or worse, having to go to the toilet, if you’re lucky you can get there…”

*SH 19*: “… before I wasn’t like this, I was… yes, I can say it, a beautiful girl, instead now this disease, its therapies, have changed everything… my body is no longer what it was, almost as if it wasn’t mine anymore”.

Theme 3: Being An Automaton

As bodily and emotional discomfort accumulated, sexuality was often described as mechanical, scheduled, and devoid of spontaneity.

Participants described a sexuality that has become increasingly scheduled, functional, and lacking in spontaneity. What was once associated with desire, complicity, and immediacy is now portrayed as a planned activity, a conscious effort to prevent sexuality from disappearing entirely from the relationship, thus emptying the physical act of its emotional meaning.

*MN 15*: “My husband, however, was close to me, he made me feel beautiful, wanted and so, right that I made an effort… but I feel that something is missing… we make love but now it is scheduled, I feel like an automaton… we feel that the feeling of those moments is being lost, it was another theft of this damn disease”.

*MN 13*: “It’s ugly to say, but it’s becoming almost like a mechanical act… I check the therapy, the progress of the last few days… in short, the romance is being lost, the spontaneity… I don’t say the desire, but almost”.

Subtheme: Reconnection

Despite these challenges, some participants described a counter-movement toward intimacy, highlighting relational resilience and the possibility of redefining closeness.

Alongside experiences of emotional distance and automatism, some participants described an opposite and unexpectedly positive process: reconnecting with their partner. In these cases, the illness was not only a challenge but also an opportunity to rediscover new forms of closeness, grounded in deep understanding, empathy, and mutual acceptance.

*NM 14*: “and that brought us closer together… I think he understood the efforts that were made and that you make even to be able to have a normal intimate life and he’s like he’s suddenly found what he’d lost… he’s got closer to me and I’m not just talking physically”.

*QJ 17*: “And now there are two of us again, ready to go on…”

In these cases, although experienced as traumatic, sexuality becomes an opportunity to strengthen the emotional bond.

Theme 4: Uncertainty About The Future

These relational and emotional tensions often led participants to question the long-term stability of their relationships.

The change in sexuality leads our participants to question their future as a couple, to identify sexuality as an important aspect of a lasting relationship and to question how this aspect might affect their relationship.

Compromised intimacy, physical discomfort, and the loss of spontaneity become warning signs of something that could undermine the stability of the couple’s relationship over time. In this context, sexuality is perceived not merely as an expression of desire or affection, but as an essential component of the relationship, an indicator of resilience and connection.

*BY 02*: “and I start to think about the future, I mean the future of the family and therefore of the couple… will I be a good partner… I mean… can she be a good partner, the one who does not seek contact with her husband? Who is ashamed of what might happen in those moments?”

EV 05: “… and this situation has created problems between us, couple problems… I don’t know if it’s right to continue being together, because now I have something less… maybe the solution could be to separate, to divorce, for his sake and maybe mine…”

*KP 11*: “The separation from my partner is ongoing and it was definitely, I don’t want to say due, I mean just because of this, but this aspect has greatly affected the couple’s life and consequently has determined our separation”.

Theme 5: Lack of communication with healthcare professionals

Across all themes, a pervasive silence from healthcare professionals emerged as a significant barrier to coping and communication

Regardless of the length of time since diagnosis, all participants complained about the lack of communication from health professionals about sexual health. A silence that stems not only from personal modesty, but from the complete lack of dedicated space for sexuality within the care pathway. Participants regarded this as a fundamental aspect of life, not only within couples, but found themselves without any point of reference, experiencing it not merely as a lack of information, but as an additional emotional barrier.

*AZ 01*: “The aspect that is frightening is the silence around this topic… no one has ever talked to me about it, no matter how much I’ve tried to open the discussion”

*FU 06*: “an important, common and essential aspect perhaps for the health of the couple but also for the mental health of those who are sick and… they don’t know about it. They talk about mental wellness, physical activity, resumption of social activities and then… You create a barrier behind the vacuum around sexuality, and I say so around the wellness of the person and the couple… why?”

*DW 04*: “… and then the fact that sexuality is not addressed by anyone creates even more problems in my opinion, no doctor, no nurse, no psychologist has ever dealt with this aspect… but why don’t they…?(legs crossed, hands clasped together, a smile on the face)… and then, I mean we all do it, it feels good and it’s normal, why be silent about something important…”

### 3.2. Summary of Participants’ Concerns

To provide a more integrated overview of the experiences described across themes, Table 3 summarizes the main disease-related and partner-related concerns. This synthesis highlights how bodily symptoms, emotional distress, and relational dynamics are deeply interconnected. To provide a clearer overview of participants’ main concerns related to the disease and to the partner, a summary table is presented (Table 3).

## 4. Discussion

Sexual health is an essential, though often overlooked, component of quality of life, particularly for individuals living with chronic illnesses such as IBD. This study explored the lived experiences of individuals with IBD about their sexuality and intimate relationships. Through in-depth interviews and phenomenological analysis, five principal themes and two subthemes emerged: the impact of the illness on sexual functioning and desire, with sub-theme stigmatization of physical symptoms leads to sexual avoidance; body image change; being an Automaton with sub-theme reconnection; uncertainty about the future and lack of communication with healthcare professionals.

These findings shed light on the complex and multifaceted impact of IBD on sexual health. The effects go beyond physical symptoms and medical treatments, extending into emotional well-being, intimate relationships, and broader psychosocial dynamics [74,75,76]. This interpretive lens is consistent with previous literature showing that variables such as abdominal discomfort, fatigue, urgency, pain, psychological distress, and treatment-related changes can profoundly affect sexual functioning and relational well-being in people with IBD [36,42].

The literature consistently shows that several IBD treatments may affect sexual function, body image, mood, and intimacy. Corticosteroids can contribute to weight gain, changes in physical appearance, and mood alterations [77,78]. Immunomodulators such as thiopurines and methotrexate have been associated with fatigue, hair loss, and reduced well-being, which may indirectly affect sexual desire and self-perception [42]. Biologic therapies, including anti-TNF, anti-integrin, and anti-IL-12/23 agents, as well as newer JAK inhibitors, may influence sexual health through their impact on inflammation, symptom control, and psychological status [77,79].

Concomitant medical and psychological conditions may also influence sexual health in IBD. Factors such as systemic inflammation, anemia, endocrine alterations, vitamin deficiencies, and fatigue can reduce libido and energy levels, while anxiety, depression, and past trauma are well-known modifiers of sexual desire, body image, and relational intimacy [42,78,80].

These are consistent with previous research showing that IBD can significantly disrupt patients’ sexual lives. Reference [81] reported that some patients completely ceased sexual activity; Michálková [74], documented sexual and relational difficulties, while Symms et al. [76], highlighted issues related to body image and lack of partner support.

The first theme emerging from this study is the impact of illness on sexual health, which appears to be a complex phenomenon involving both physical and psychological dimensions. Participants indicated that sexuality is a central part of their couple life, but is profoundly affected by the disease. Not only physical changes (e.g., sexual dysfunction), but also emotions like shame contribute to emotional and physical detachment from their partner [82].

The stigmatization of physical symptoms led to strong sexual avoidance, resulting in loss of intimacy and emotional contact. In particular, shame about symptoms, such as odors, leakage, and noises, was perceived as a major barrier to intimacy, leading to the avoidance of sexual activity, generated by shame and fear of partner judgment or embarrassment during physical contact [83], common sentiments among patients with IBD [84,85].

Alterations in sexual health are more common in patients with IBD than in the general population and are a key determinant of quality of life [78,86,87].

A higher prevalence of sexual health impairments has been reported in external epidemiological studies, particularly among women and individuals with active disease or perianal involvement [39,86]. Estimates from previous research indicate that 45–60% of women and 15–25% of men with IBD experience some degree of sexual dysfunction, rates substantially higher than those observed in the general population [47,88,89]. These figures refer to published data and are provided here solely to contextualize the broader literature, not as results of the present qualitative study.

Furthermore, compared to the general population, people with IBD tend to report lower levels in several aspect of sexual function. For men, the most impaired areas include erectile function, sexual desire, orgasm, satisfaction, and overall quality of sexual activity; in women, desire, arousal, lubrication, orgasm, pain during intercourse, satisfaction, and perceived quality are more impacted [90]. It has also been shown that impairments in sexual health, particularly erectile dysfunction, are not necessarily associated with the objective clinical state of the disease, but appear more frequently related to the presence of subjective symptoms and psychological factors such as anxiety and depression [81,86].

In the literature, changes in sexual health among people with IBD are described as multifactorial, involving physical symptoms (such as abdominal pain, fatigue, diarrhea, incontinence) as well as psychological factors like anxiety, depression, and distorted body image [39,78,91].

Another emerging theme is the change in body image, perceived as one of the most painful experiences. Patients reported an ongoing struggle and increasing dissatisfaction with their body, perceived as “foreign” and “altered.” Weight gain and other physical changes made them feel less attractive and often unable to recognize themselves. The sensation of “no longer being themselves” directly impacted how they experienced sexuality, contributing to a loss of desire and reduced sexual spontaneity.

The body image of patients with IBD is profoundly affected by the physical changes caused by both the disease itself and the treatments used. In particular, drugs such as corticosteroids can cause noticeable and often experienced changes with discomfort, such as weight gain, abnormal distribution of body fat, acne, and facial rounding, which contribute to a worsening of self-perception and self-esteem [39,78,86].

Other immunosuppressive therapies can also negatively affect appearance: for instance, azathioprine and 6-mercaptopurine can increase sensitivity to sunlight, methotrexate is linked to hair loss, and anti-TNF-α biological drugs have been associated with the development of psoriasis [92,93,94,95].

During periods of disease activity, patients may present with visible symptoms such as marked weight loss, hair loss, and skin changes. In some forms, such as in Crohn’s disease, particularly when perianal involvement is present, lesions and fistulas may occur in the perineal area, significantly affecting body image and psychological well-being [96,97]. Extraintestinal comorbidities, particularly cutaneous manifestations, may further exacerbate the impact of inflammatory bowel disease on patients’ sexual lives. Dermatological involvement is among the most frequent extraintestinal manifestations of IBD and includes conditions such as erythema nodosum, pyoderma gangrenosum, psoriasis-like lesions, acneiform eruptions, and therapy-related skin changes [98,99,100]. These manifestations are often visible and difficult to conceal, directly affecting physical appearance and contributing to heightened self-consciousness, shame, and reduced self-esteem. Previous studies have highlighted that skin involvement in IBD is associated with psychological distress, impaired quality of life, and social withdrawal [98,99]. In the context of sexuality, visible skin changes may intensify feelings of reduced attractiveness and fear of negative evaluation by one’s partner, thereby promoting sexual avoidance and emotional distancing. Although cutaneous manifestations were not always explicitly verbalized by participants in our study, they may act as silent amplifiers of body image disturbance, reinforcing the perception of a “changed” or “unreliable” body already compromised by the disease. These findings support the importance of considering dermatological comorbidities as an integral component of the sexual and relational experience of people living with IBD and underscore the need for a holistic, multidisciplinary approach to care that addresses both physical appearance and sexual well-being [98,100].

The third theme that emerged concerns sexuality as sometimes experienced mechanically. Lack of spontaneity, the need for planning, and the sensation of performing a “duty” rather than sharing an experience were mechanisms adopted to maintain the relationship. Although there was a desire to maintain intimacy, many described how feelings and passion gradually fading. Some patients reported that intimacy had become an “automatic” gesture, a duty to preserve the relationship, as also reported in the literature, where even when intimacy is granted to please the partner, it risks being experienced automatically and without personal satisfaction [83]. This behavior may serve as a defense mechanism, but it risks harming sexual satisfaction and the strength of the bond over time. Particularly among women, as [101] report, sex is considered a “marital duty,” generating a strong sense of guilt when illness prevents sexual intercourse.

However, in some couples, a “reconnection” occurred, where communication and mutual understanding helped overcome difficulties, leading to renewed emotional closeness. Reference [102] reveals how IBD can affect couples’ relationships in an ambivalent way. For some couples, it can create distance and cause difficulties, interfering with life plans and generating emotional fatigue for both the patient and their partner. In other cases, however, experiencing the disease can encourage greater openness in communication and strengthen the emotional bond, creating new forms of intimacy based on empathy and mutual understanding.

Uncertainty about the future emerged as a critical element. The fear of no longer being a “good” partner, anxiety about no longer being able to sexually satisfy the partner, fear of abandonment, and loss of shared plans were widely reported. As shown in studies by [103,104], the fear of being unable to experience fulfilling sexuality leads to feelings of vulnerability and loneliness, emphasizing sexuality as a cornerstone of the relationship.

A common theme across all interviews was the lack of communication with healthcare professionals. Despite sexuality being central to couple relationships, none of the participants had received information or support from healthcare providers regarding their sexual life after diagnosis. Feeling ignored on such a sensitive issue created an additional barrier, with patients feeling abandoned regarding an aspect crucial to their well-being. This silence contributed to increased frustration, isolation, and the sense that their sexual health was considered secondary. Literature confirms that sexuality among patients with IBD is rarely addressed by healthcare professionals [105,106] despite international recommendations for a holistic approach to care [76,107]. Indeed, Marín et al. [78] reported that 64% of women and nearly 50% of men with IBD expressed a desire to receive information about the impact of the disease on their sexuality at the time of diagnosis. However, as also highlighted by Rasmussen Edelbo et al. [108], sexuality remains an unaddressed topic, often perceived as taboo by both patients and healthcare professionals. In the same study, 86% of patients stated they had never discussed sexual issues with medical staff. Among them, 38% wished the topic had been raised by healthcare providers, 34% considered it a taboo subject, and 25% found it difficult to talk about openly [108]. These findings highlight the need for more attentive and comprehensive care that includes sexual health as a key part of managing patients with IBD. Unfortunately, physicians treating IBD often lack the training and guidance to create safe spaces for discussing this private subject and to properly include the assessment and treatment of sexual well-being in their practice [106].

From a clinical perspective, a pragmatic and evidence-informed approach to sexual health in IBD may include routine, sensitive screening, either through one or two targeted questions or, when appropriate, validated tools such as the FSFI or IIEF. When concerns emerge, clinicians should consider potentially reversible contributors, including uncontrolled disease activity, anemia, pain, fatigue, psychological distress, and medication-related adverse effects. Management may involve optimizing disease control, addressing mood symptoms, reviewing pharmacological regimens, or referring patients to pelvic floor physiotherapy or specialized sexual therapy when indicated. In addition, discussions about contraception and fertility are essential, particularly in the context of pre-conception planning for individuals receiving thiopurines, biologics, or JAK inhibitors. Integrating these elements into routine care can help clinicians address sexual health more systematically while remaining aligned with person-centered IBD management.

Creating a safe and non-judgmental space where patients can express their sexual concerns promotes not only relational and emotional well-being but also trust in the care process. Strategies such as motivational interviewing have proven effective in improving therapeutic adherence and the active involvement of patients in disease management [109], with potential benefits also in addressing intimate and sensitive issues such as sexual health.

Patients hope for professional support extended to their partners, believing that greater information could improve adaptation and sexual well-being [44,110,111]. As highlighted in this study, the lack of information creates an assistance gap. Educating partners as well as patients enhances sexual life quality. In the qualitative study by Altschuler et al. [112], partners who were well-informed and supportive did not experience sexual problems postoperatively, whereas patients whose partners were unsupportive experienced a negative impact on sexual life. Thus, patients with IBD and their partners must express sexual concerns and receive the necessary support. A primary therapeutic strategy for improving sexual health is the attention healthcare professionals dedicate to sexuality. This study aimed to explore and deeply understand the lived experiences of individuals with IBD about sexuality and intimacy, highlighting the importance of acknowledging these aspects as an integral part of the patient’s experience. Promoting authentic and non-judgmental communication among patients, partners, and healthcare professionals can significantly help improve the quality of life for those living with IBD.

These findings also carry important implications for clinical practice, particularly regarding communication, patient education, and the integration of sexual health into routine IBD care. Sexual health is a fundamental component of quality of life, yet it remains insufficiently addressed in clinical encounters due to cultural taboos, limited training, and discomfort among healthcare professionals. A more holistic and person-centered approach is therefore needed, one that systematically incorporates sexuality into assessment, dialogue, and care planning.

Participants consistently reported a lack of open, informed communication about sexuality, which contributed to feelings of shame, uncertainty, and avoidance. Healthcare professionals can play a key role in addressing this gap by creating a safe, non-judgmental environment for discussing sexual concerns and by providing clear, evidence-based information on how IBD and its treatments may affect sexual function, body image, and fertility. Enhancing communication skills and relational sensitivity within clinical teams may help normalize these conversations and reduce the emotional burden experienced by patients.

Emotional support also emerged as a critical unmet need. Many participants described feelings of inadequacy, loss of desirability, and emotional withdrawal. Healthcare professionals trained in active listening and relational competence can help patients process these emotions and, when appropriate, facilitate access to psychological or sex-therapy services. The involvement of partners is equally important: shared understanding and open communication can strengthen the couple’s resilience, whereas lack of dialogue may deepen emotional distance. Couple-focused education or supportive interventions may therefore contribute to restoring intimacy and relational balance.

Finally, the findings highlight the need for structured training programs on sexual health communication for all professionals involved in IBD care. Cultural barriers, lack of confidence, and insufficient preparation were identified as major obstacles to addressing sexuality in clinical practice. Training that emphasizes communication, cultural competence, and relational sensitivity would enable professionals to engage proactively and respectfully with this dimension of care, ultimately improving the quality of life of individuals living with IBD.

### Limitations and Strengths

This study has several limitations that affect the interpretation and generalizability of the results. First, the predominance of female participants may have influenced the findings, as the literature shows that women tend to report sexual problems more frequently compared to men and may also be more inclined to express emotional and physical difficulties related to sexuality. Moreover, the variability in the time since diagnosis may have influenced participants’ perspectives, as individuals with a longer history of illness might have engaged in personal reflection and adaptation processes. In addition, differences in the clinical stage of the disease (e.g., active symptoms vs. remission) at the time of the interview represent a further limitation, as symptom severity may significantly impact the experience of sexuality. As well as all participants were recruited from the same region in Southern Italy, a context characterized by specific cultural, social, and religious influences that may have shaped how individuals experienced and narrated their sexuality. In more traditional settings, topics related to intimacy and sexual health may still be perceived as taboo, potentially affecting participants’ willingness to discuss these aspects openly and influencing the language used to describe their experiences. Therefore, the cultural homogeneity of the sample represents an additional limitation to the generalizability of the findings, which may not fully capture the experiences of patients from different cultural backgrounds or geographical areas.

An additional limitation is the absence of detailed clinical variables, including specific pharmacological regimens, surgical history, ostomy status, and concomitant medical or psychological conditions that may influence libido. These data were not systematically collected because the phenomenological design prioritized lived experience over clinical correlations, which reduces the possibility of examining how such factors may have shaped participants’ accounts.

Among the strengths of the study is the in-depth analysis of patients’ individual experiences, which captures the complexity and depth of sexual issues related to living with a stoma. This qualitative approach allows for a more nuanced understanding of the emotions, perceptions, and behaviors associated with sexuality, providing meaningful insights for a more targeted and personalized management of the psychological and physical needs of patients.

## 5. Conclusions

This study explored the lived experience of sexuality and intimacy in patients with IBD, highlighting the impact of the disease on these dimensions and underscoring how sexual health is closely linked to emotional well-being and overall quality of life.

These insights highlight the need to interpret sexual and relational experiences within both their social context and the clinical realities of IBD, including symptom burden, treatment effects, and disease chronicity.

These issues demonstrate the complexity of this experience and the importance of sexuality in patients’ quality of life. It is crucial to address the silence surrounding these issues by incorporating sexual health into the care pathway through a multidisciplinary, person-centred approach. Training professionals and involving partners are key strategies for improving care and patients’ overall well-being.

## Figures and Tables

**Table 1:** Socio-demographic and clinical characteristics of participants.

Code	Gender	Age	Marital Status	Diagnosis (CD/UC)	Years Since Diagnosis	Education Level
AZ 01	F	31	Married	UC	9	High school
BY 02	F	27	Single	UC	4	University
CX 03	M	25	Married	CD	2	University
DW 04	F	44	Married	CD	19	University
EV 05	F	34	Married	UC	11	University
FU 06	M	37	Married	CD	18	High school
GT 07	M	55	Married	UC	31	High school
HS 08	F	42	Married	CD	16	Middle school
IR 09	M	51	Married	CD	28	High school
JQ 10	M	38	Married	UC	14	High school
KP 11	F	37	Married	CD	10	University
LO 12	F	48	Married	UC	26	High school
MN 13	M	45	Married	UC	24	Middle school
NM 14	F	51	Married	UC	31	High school
OL 15	F	43	Married	CD	18	Not reported
PK 16	M	28	Single	CD	12	University
QJ 17	M	53	Married	UC	26	Middle school
RI 18	M	47	Married	UC	19	High school
SH 19	F	45	Married	CD	22	Middle school

**Table 2:** Themes and descriptions from the qualitative analysis.

Theme	Subtheme
Impact of the disease on sexuality	Stigmatization of physical symptoms leading to sexual avoidance
Body image changes	
Being an automaton	Reconnection
Uncertainty about the future	
Lack of communication with healthcare professionals	

**Table 3:** Main concerns related to disease and partner.

Disease-Related Concerns	Partner-Related Concerns
Unpredictability of the disease course	Fear of undermining the foundations of the relationship
Stigmatization of physical symptoms (incontinence, bloating, odors, noises)	Avoidance of physical and sexual contact with the partner
Shame and embarrassment related to bodily symptoms	Fear of partner judgment and embarrassment during intimacy
Altered body image and feeling of a “foreign” body	Fear of no longer being attractive or desirable for the partner
Weight fluctuations, scarring, fear of a possible stoma	Anxiety about partner’s sexual interest
Reduced sexual desire and emotional distress	Sexuality experienced as a duty to preserve the relationship
Sexuality perceived as mechanical and scheduled (“being an automaton”)	Loss of spontaneity and emotional closeness
Emotional detachment and self-alienation	Risk of emotional distance within the couple
Uncertainty about disease progression and future	Uncertainty about relationship stability and fear of separation
Lack of communication with healthcare professionals about sexuality	Lack of guidance to support couple communication

## Data Availability

The data presented in this study are not publicly available due to ethical and privacy restrictions.

## Abbreviations

The following abbreviations are used in this manuscript:

IBD	Inflammatory Bowel Disease
CD	Crohn’s Disease
UC	Ulcerative Colitis
WHO	World Health Organization
